# Microstructure Characteristics, Mechanical Properties and Strain Hardening Behavior of B2 Intermetallic Compound-Strengthening Fe-16Mn-9Al-0.8C-3Ni Steel Fabricated by Twin-Roll Strip Casting, Cold Rolling and Annealing

**DOI:** 10.3390/ma16155417

**Published:** 2023-08-02

**Authors:** Baoguang Zhang, Kun Yang, Xiaoming Zhang, Haitao Liu, Weina Zhang, Jian Wang

**Affiliations:** 1 State Key Laboratory of Rolling and Automation, Northeastern University, Shenyang 110819, China; 2 State Key Laboratory of Porous Metal Materials, Northwest Institute for Nonferrous Metal Research, Xi’an 710016, China

**Keywords:** lightweight steel, B2 intermetallic, twin-roll strip casting, tensile properties, gradient microstructure

## Abstract

In this study, the Fe-16Mn-9Al-0.8C-3Ni (wt.%) lightweight steel was fabricated by novel twin-roll strip casting technology. The microstructure, tensile properties and strain-hardening behavior of the present steel have been investigated and compared to those of conventionally processed steels with similar chemical compositions. After annealing, a unique gradient microstructure of intermetallic compound (B2)-austenite was obtained along the thickness direction, consisting of granular B2 (average: 430 nm) and fine austenite (average: 1.82 μm) at the surface layer, blocky B2 (average: 1.03 μm) and medium austenite (average: 3.98 μm) at the quarter layer and polygonal B2 (average: 1.94 μm) and coarse austenite (average: 6.13 μm) at the center layer. The cooperative action of B2 pinning dislocation, plane slip and back stress led to stronger strain hardening, among which the strong back stress effect originated from the multistage discontinuous austenite deformation and the mechanical incompatibility between austenite and B2 is believed to be the most important reason, thereby achieving an excellent balance of strength (ultimate tensile strength: 1147 MPa) and ductility (total elongation: 43.2%). This work not only developed a new processing way to fabricate Ni-containing Fe-Mn-Al-C lightweight steel with outstanding mechanical properties, but also provided a potential solution for manufacturing some other metallic materials accompanied by brittle B2 intermetallic.

## 1. Introduction

Recently, Fe-Mn-Al-C lightweight steels have attracted much attention due to their lightweight potential as well as their superior comprehensive mechanical properties [1,2,3,4,5,6]. As a new strategy, Kim et al. introduced B2 intermetallic to improve the strength–ductility balance of lightweight steels (Fe-15Mn-10Al-0.8C-5Ni, wt.%) by utilizing their nonshearable nature [7]. The B2 phase (such as FeAl, TiAl and NiAl) is an intermetallic compound phase that shows an ordered simple cubic lattice of the CsCl type, whose space group is Pm3m. The Fe/Ti/Ni and Al atoms occupy the two superlattices of this structure, respectively. As a hard, brittle intermetallic compound, B2 particles show poor plasticity at ambient temperature in the bulk state but offer an attractive combination of physical and mechanical properties such as low density and good corrosion, oxidation and/or wear resistance [7]. B2 intermetallic with different morphologies has generally been observed in these steels, including elongated bands along the rolling direction (width of 1–20 μm), polygonal-shaped particles at austenite boundaries (size of 200 nm–7 μm) and fine particles inside austenite grains (size of 20–700 nm) [7,8,9,10,11,12]. Park et al. reported that the presence of elongated B2 bands can lead to an adverse effect on formability, such as bendability and hole expansion ratio [9]. To eliminate B2 bands, lightweight steel with higher alloying elements (Fe-21Mn-10Al-1C-5Ni, wt.%) has been invented by Hwang et al., showing a good balance of strength and ductility [13]. However, it will inevitably increase the cost of raw materials. Moreover, the addition of higher Mn and C favors the formation of deleterious particles (e.g., κ-carbides) along grain and interphase boundaries [1,13], which will seriously deteriorate the ductility. Thus, it is necessary to explore a new way to eliminate B2 bands.

Furthermore, although high Al addition is beneficial for reducing weight, it will lead to various difficulties in the processing of lightweight steels. For example, Al can react strongly with the mold flux during casting, leading to a change in the composition of the mold flux and, accordingly, a deterioration of its performance [14,15,16]. Further, this reaction causes alumina formation in the melt, resulting in a clogging problem during continuous casting [17,18,19]. In addition to these difficulties in the casting process, Al addition can also deteriorate the subsequent hot and cold workability of high-Al lightweight steels via the formation of κ-carbide particles [20,21,22].

The above-mentioned difficulties make twin-roll strip casting (TRSC) an effective technology for fabricating high-Al lightweight steels. Twin-roll strip casting is a novel forming technology with a near-net shape, directly producing a few millimeter-thick sheets (1–5 mm) from molten metal due to subrapid solidification (10^2^–10^4^ °C/s) [23]. This forming technology has been widely utilized for the plate/strip production of magnesium alloys [24,25], aluminum alloys [26,27], low carbon steels [28,29], silicon steels [30,31] and TWIP/TRIP steels [32,33]. The most important characteristic of TRSC is that it can provide a fast cooling rate during casting, leading to subrapid solidification of the molten steel. This will inevitably affect the microstructure (especially the B2 intermetallic) and, accordingly, the mechanical properties of Ni-containing Fe-Mn-Al-C lightweight steels.

In this work, we attempted to utilize the twin-roll strip casting technology as a new method for manufacturing a Ni-containing Fe-Mn-Al-C lightweight steel (Fe-16Mn-9Al-0.8C-3Ni, wt.%) so as to eliminate B2 bands and acquire fine B2 particles. In order to decrease the size and quantity of B2 intermetallic to achieve good ductility, 9% Al and 3% Ni were selected. The main focus of the current work was on microstructure evolution and resultant tensile properties, which were compared to those of conventional casting ingots of similar composition. In addition, the deformation behavior and strain hardening behavior were investigated in detail as well.

## 2. Materials and Methods

**Figure 1:**
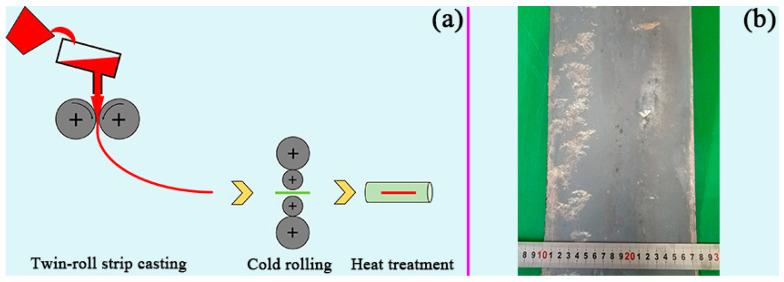
Schematic diagram showing the applied processing route in fabrication of experimental steel (**a**) and real photograph of as-cast strip (**b**).

The investigated Ni-containing lightweight steel with a nominal composition of Fe-15.8Mn-9.1Al-0.78C-3.3Ni (in wt.%) was fabricated by a vertical type of twin-roll strip caster. The present steel (30 kg) was melted in a vacuum induction furnace, and the molten steel was poured into a preheated tundish to flow through a nozzle under Ar_2_ shielding into the water-cooled casting rollers. The speed of strip casting was set to be 0.26 m/s and the initial roll gap was set to be 1.9 mm to obtain complete and well-shaped strips. The final thickness of the cast strips was measured to be 2.02 mm. Then, the as-cast strip was directly cold-rolled to 1 mm. After that, the cold-rolled sheets were annealed at 900 °C for 15 min under an argon atmosphere and water-quenched to room temperature. The schematic diagram of the processing route for the present steel is shown in Figure 1a. The real photograph of an as-cast strip created from twin-roll strip casting is presented in Figure 1b.

The typical microstructure was characterized by optical microscope (OM) and scanning electron microscope (SEM, QUANTA 600, FEI, Hillsboro, OR, USA) equipped with electron backscattered diffraction (EBSD) technology. B2 particles were observed using the transmission electron microscopy (TEM, Tecnai G^2^ F20, FEI, Hillsboro, OR, USA) at an operating voltage of 200 kV. The tensile specimens with a gauge length of 25 mm were machined parallel to the rolling direction. Tensile test at room temperature was performed on an electronic universal testing machine (WDW-50kN, Changchun Kexin Testing Instrument Co., Ltd., Changchun, China) with a strain rate of 10^−3^ s^−1^.

## 3. Results

### 3.1. As-Cast Microstructure

**Figure 2:**
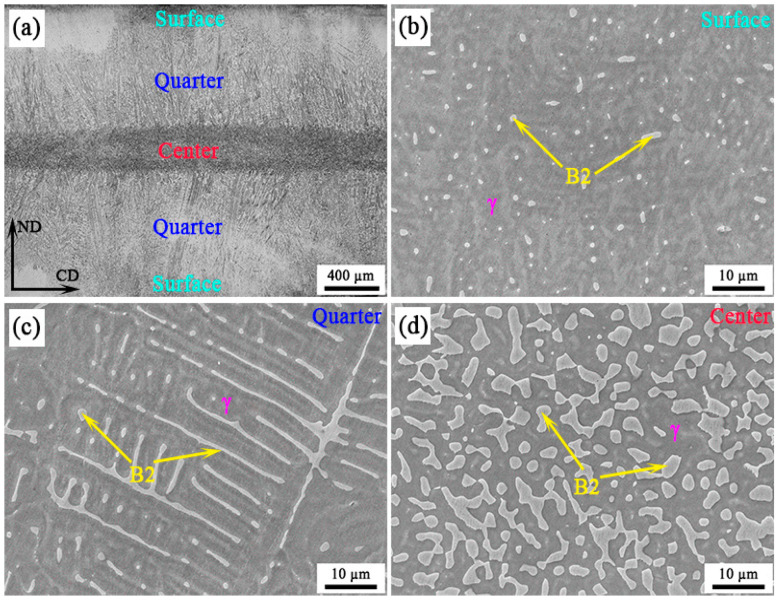
Microstructure of experimental steel in as-cast condition: OM micrograph of through-thickness characteristics (**a**) and high magnification SEM micrograph of surface layer (**b**), quarter layer (**c**) and center layer (**d**). (CD: casting direction, ND: normal direction, γ: austenite.)

**Figure 3:**
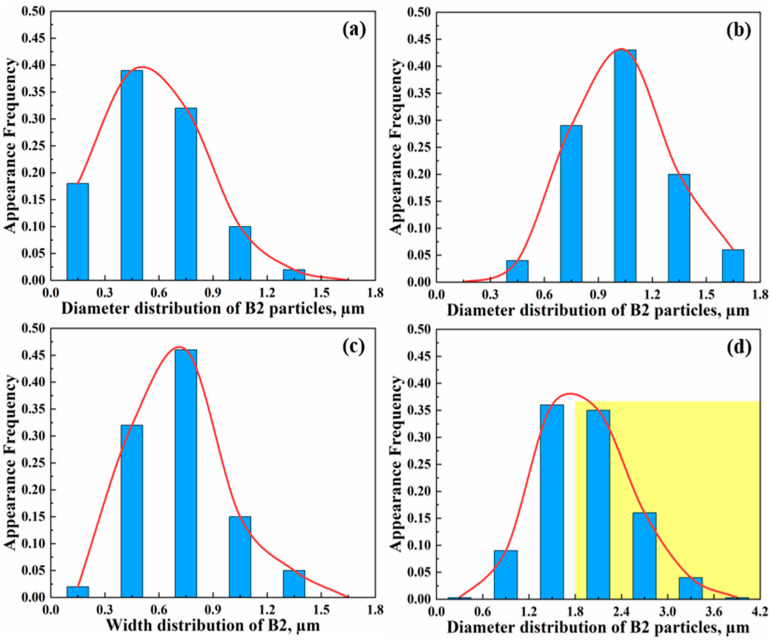
Diameter and width distribution of B2 at different layers in the as-cast sample: (**a**) surface layer; (**b**,**c**) quarter layer; (**d**) center layer. (The yellow shadow in d is for the convenience of data comparison).

Figure 2a shows the representative OM micrograph of an as-cast strip along the thickness direction, exhibiting a typical gradient microstructure consisting of five sections with different characteristics of B2 intermetallic, namely, two superficial fine equiaxed crystal zones (surface layer), two columnar crystal zones (quarter layer) and one central coarse equiaxed crystal zone (center layer). At the surface layer, B2 was characterized as fine granular particles (Figure 2b), showing an average diameter mostly below 0.9 µm (Figure 3a). At the quarter layer, B2 with two different morphologies can be observed, namely granular and strip-like (Figure 2c). The average diameter of granular B2 particles is mainly concentrated in the range of 0.6–1.5 µm, which is obviously coarser than that in the surface layer (Figure 3b). Moreover, B2 particles with a size smaller than 0.3 µm cannot be found at all, while the appearance frequency of B2 particles larger than 1.5 µm significantly increases to 0.06 from 0. Additionally, strip-like B2 shows an average width of 0.72 µm, and mostly concentrates between 0.3 and 1.2 µm (Figure 3c). With regard to the center layer, B2 particles present a polygonal morphology, and their size further increases significantly (Figure 2d and Figure 3d). Most (>50%) of polygonal B2 particles are larger than 1.8 µm (marked by the yellow rectangle in Figure 3d), which has not been observed in surface and quarter layers. Additionally, it can be calculated that the width of the quarter layer through the total thickness of the annealed sheet is approximately 800 µm, accounting for 80%. Additionally, the surface and center layers cover 7% and 13%, respectively.

**Figure 4:**
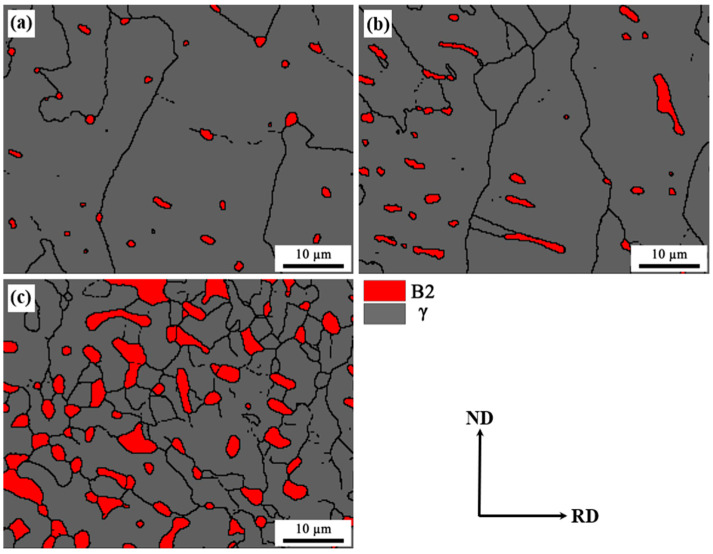
EBSD phase maps of as-cast steel: (**a**) surface layer; (**b**) quarter layer; (**c**) center layer.

EBSD phase maps of different layers are presented in Figure 4, showing that B2 particles distribute in the austenite matrix homogeneously and dispersedly. The B2 phase can be observed at the interior and grain boundaries of austenite. On the contrary to the B2 phase, the austenite grain size at the surface layer (average: 42.3 µm) is the largest, which is nearly six times larger than that of austenite at the center layer (average: 7.31 µm).

### 3.2. As-Annealed Microstructure

**Figure 5:**
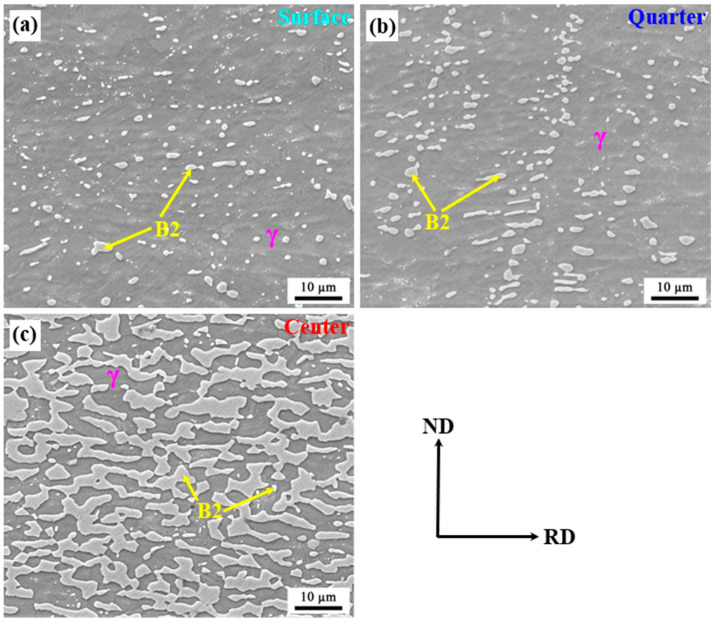
SEM images of as-annealed sample: (**a**) surface layer; (**b**) quarter layer; (**c**) center layer.

**Figure 6:**
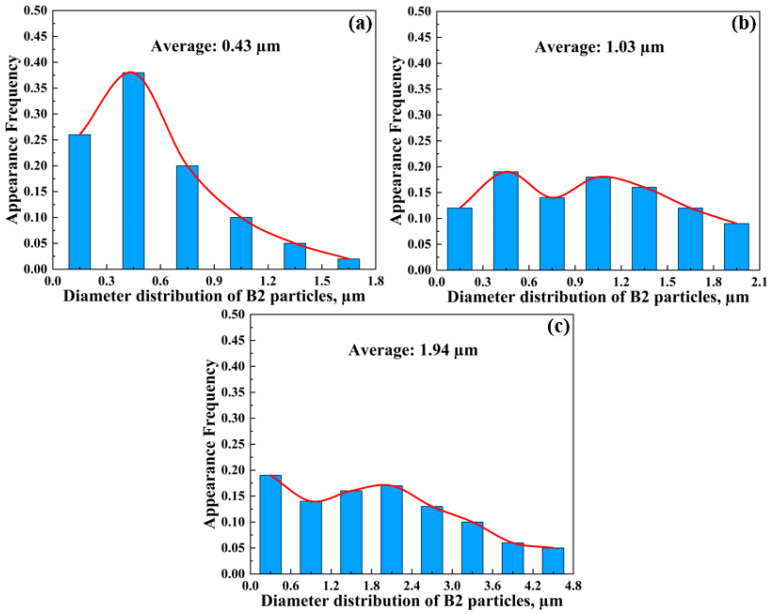
Size distribution of B2 at different layers in the as-annealed sample: (**a**) surface layer; (**b**) quarter layer; (**c**) center layer.

The representative microstructure of the as-annealed sample shows that the gradient microstructure is still inherited after cold rolling and annealing compared with the as-cast condition (Figure 5). Additionally, the number density of B2 increases significantly no matter which layer is used (Figure 2 and Figure 5). At the surface layer, the morphology of B2 was still primarily characterized as a granular shape. The appearance frequency of B2 smaller than 0.3 µm increases to 0.26 from 0.18 (Figure 6a), indicating the precipitation of new granular B2. Additionally, B2 with a size between 1.5 and 1.8 µm can be observed in annealed samples but not in as-cast samples. At the quarter layer, the aspect ratio of B2 decreases greatly, meaning that strip-like B2 transforms into a short rod with a blocky shape after cold rolling and annealing. Moreover, the appearance frequency of B2 smaller than 0.3 µm significantly increases to 0.12 from 0, and some B2 larger than 1.8 µm can be observed (Figure 6b). At the center layer, the quantity of polygonal B2 smaller than 0.6 µm exhibits a sharp increase, while the appearance frequency of B2 with a size between 1.2 and 2.4 µm dramatically decreases from 0.71 to 0.33. Furthermore, the presence of coarse polygonal B2 particles larger than 4.2 µm can be confirmed in annealed samples (Figure 6c). No matter which layer, the cold rolling and annealing cannot only increase the quantity of B2 in the minimum size range (0–0.6 µm), but also promote the formation of larger B2. This result indicates that the size distribution of B2 in annealed steel is more uniform than that in as-cast steel.

Note that no B2 bands with a high aspect ratio and a length longer than 10 µm can be found in the present annealed steel compared with other conventionally processed Ni-containing Fe-Mn-Al-C lightweight steels with the same chemical composition [7,8]. It has been demonstrated that the brittle, coarse B2 bands will lead to poor formability and ductility [9,13]. This result suggests that the twin-roll strip casting technology can effectively inhibit the formation of B2 bands, so it is very promising to achieve the purpose of optimizing the mechanical properties of Fe-Mn-Al-C lightweight steels accompanied by brittle B2 intermetallic. More interestingly, the gradient microstructure after final annealing has never been reported in other conventionally processed Ni-containing Fe-Mn-Al-C lightweight steels, which will inevitably have a significant effect on the mechanical properties.

**Figure 7:**
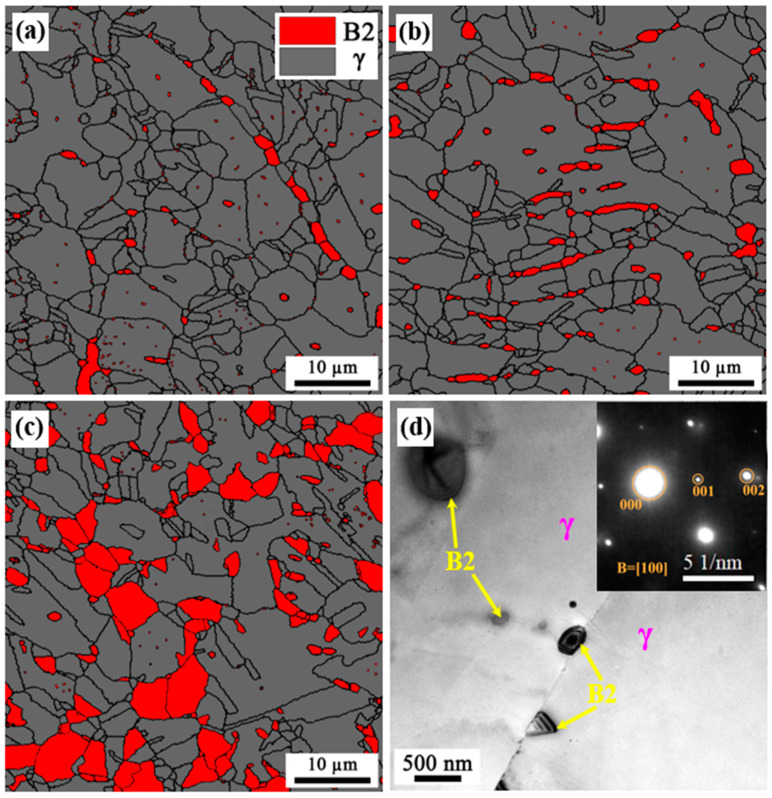
Microstructure of the as-annealed sample: EBSD phase map: surface layer (**a**), quarter layer (**b**) and center layer (**c**); TEM map and SADP from B2 particle (**d**).

In order to characterize the as-annealed microstructure in more detail, EBSD and TEM analyses were employed with respect to the distribution of B2 and austenite. The EBSD phase maps at different layers of the annealed sample are presented in Figure 7a–c, showing the B2-austenite duplex microstructure with obvious grain and phase boundaries. It can be observed that B2 particles not only distribute at austenite grain boundaries, but also form inside austenite grains. The formation of intergranular B2 is attributed to its nucleation and growth at the grain boundaries of recrystallized austenite. Additionally, the intragranular B2 particles usually precipitate at deformation shear bands of unrecrystallized austenite [7]. Furthermore, a TEM bright-field image showing intergranular and intragranular B2 particles is presented in Figure 7d. Additionally, the superlattice spots of SADP in the inset can further confirm the formation of B2 particles at the grain boundaries and the interior of austenite.

**Figure 8:**
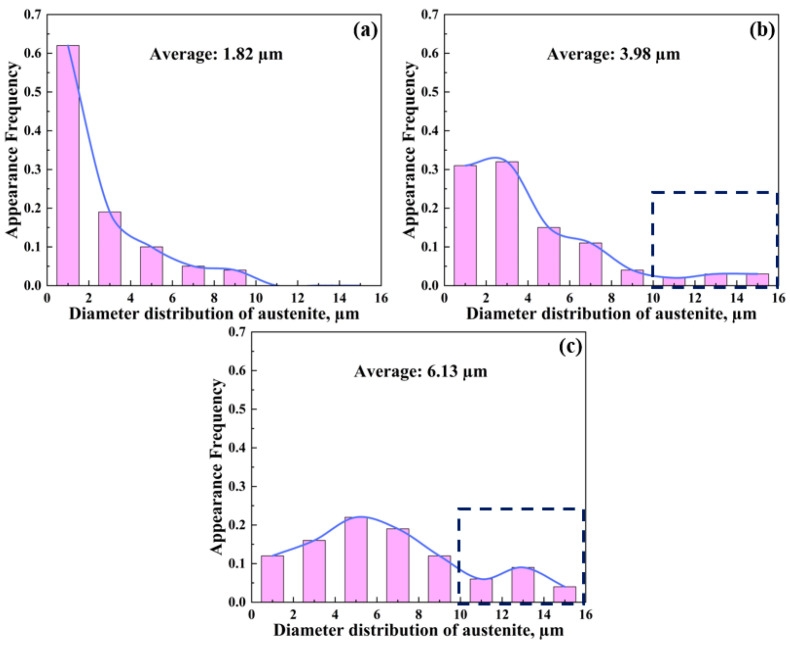
Size distribution of austenite at different layers in as-annealed sample: (**a**) surface layer; (**b**) quarter layer; (**c**) center layer.

The diameter distribution of austenite at different layers was calculated as well (Figure 8), showing the same layered gradient microstructure as B2. The size of the austenite at the surface layer is less than 10 µm (Figure 8a). As we move closer to the center layer, austenite with a size of 10–16 µm can be observed (Figure 8b,c), and the appearance frequency increases significantly. The average diameter of the austenite increases from 1.82 µm at the surface layer to 6.13 µm at the center layer. It is believed that the austenite gradient microstructure will make an additional contribution to mechanical properties. Moreover, the average size of all austenite grains has been calculated as 4.52 µm, which is nearly three times larger than that reported by Kim [7] and Hwang [13]. The coarse austenite matrix may be beneficial to the excellent ductility.

### 3.3. Mechanical Properties

**Figure 9:**
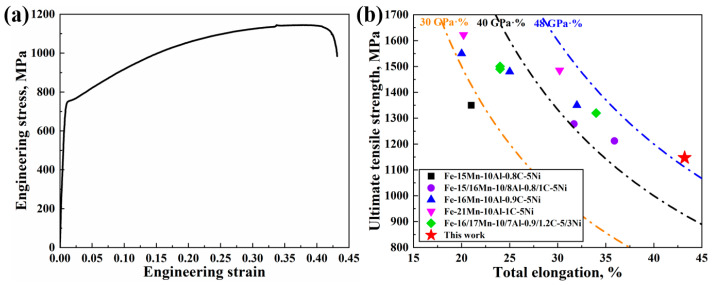
Engineering stress-strain curve of as-annealed sample (**a**) and comparison of tensile properties between present steel and other Ni-containing Fe-Mn-Al-C lightweight steels with conventional ingot casting technology [7,9,13,34,35] (**b**).

Figure 9a shows the room-temperature tensile curves of the as-cast and as-annealed steels. It can be found that the yield strength (YS), ultimate tensile strength (UTS) and total elongation (TEL) of as-cast steel are 625 MPa, 866 MPa and 34.7%, respectively. Additionally, the YS, UTS and TEL of the present annealed steel increased to 760 MPa, 1147 MPa and 43.2%, respectively. Surprisingly, the product of UTS and TEL (UTS×TEL) of present annealed steel can reach as high as 49.6 GP%, indicating an excellent strength–ductility balance. The microhardness of steels after casting, cold rolling and heat treatment has been measured to be 278 ± 21 HV, 523 ± 18 HV and 416 ± 24 HV, respectively. The comparison of tensile properties between the present annealed steel and other conventionally processed Ni-containing Fe-Mn-Al-C lightweight steels is shown in Figure 9b. In general, the Ni-containing Fe-Mn-Al-C lightweight steels show a high YS due to the nonshearable nature of B2. However, the brittleness of B2 may result in a low TEL for these steels. To our best knowledge, up to now, no Ni-containing Fe-Mn-Al-C lightweight steels with a frequently used strain rate (10^−3^ s^−1^) have been published showing a TEL higher than 40% as well as a UTS higher than 1100 MPa. Such excellent mechanical properties of the present steel are probably related to the special gradient microstructure produced by twin-roll strip casting technology.

## 4. Discussion

### 4.1. Microstructure Difference between Present Steel and Conventionally Processed Steel

The most notable observation is the gradient microstructure of as-cast and as-annealed steels, containing B2 particles and austenite with different size distributions. Since the chemical composition of the present steel is similar to other Ni-containing Fe-Mn-Al-C lightweight steels [8,13,35,36,37], the microstructure differences are believed to originate from the different manufacturing processes. During the casting process, the molten metal contacts the casting rollers directly, which leads to great undercooling [38]. The nonequilibrium solidification results in the formation of austenite at the surface layer [39]. Then, some refined granular B2 can nucleate concurrently and grow with austenite. Even though twin-roll strip casting technology has the characteristics of subrapid solidification (102–105 °C/s), the cooling rate gradient can still form along the thickness direction during the solidification process [38,40,41]. The cooling rate gradually decreases from the surface to the center of the as-cast strip, resulting in a difference in microstructure along the strip thickness. At the quarter layer, with the increase in strip thickness, the solidification velocity of molten steel decreases, and thus austenite with columnar dendrite morphology forms. The casting rollers are always in close contact with the casting strip during the casting process to ensure that the direction perpendicular to the surface of the casting roll has a fast heat dissipation rate, which is conducive to the optimal growth of small grains at the surface layer near the liquid in its opposite direction, of which the growth rate is the fastest in the direction of the primary crystal axis, so these grains rapidly grow side by side in the liquid phase to form the columnar crystal, and the latent heat of crystallization and the heat of the liquid steel itself are quickly taken away [42]. Then, the columnar crystals will bifurcate, forming dendrites due to the small temperature gradient. The dendrites exhibited an inclination angle of 5–20° from the normal direction of the as-cast strip, and this can mainly be attributed to the extrusion force on the strip originating from the caster roller during the strip casting process [41]. The strip-like and rod-like B2s are located at the core of the primary or secondary dendrite because of the uncompleted transformation of the primary δ-ferrite to austenite during subsequent solidification. The formation of primary δ-ferrite in Fe-Mn-Al-C-Ni steels has been confirmed by Hwang [13] and Zhang et al. [43]. After that, the heat transfer coefficient on the strip surface decreases when the strip leaves the kiss point of the two rolls, so the preferential growth of free crystals ahead of the dendritic solidification front will grow by means of equiaxed grain to form the center layer [39]. The slower cooling rate can provide more elements (such as Al and Ni) to form the B2 phase since low partition coefficients (<1) of Al and Ni elements [44] will be discharged into the melt at the center layer [45], resulting in the growth of the B2 particles, which are obviously coarsened by 2–3 times compared with those at the surface layer (Figure 3).

After that, although the as-cast strip was subsequently cold-rolled and annealed, it still inherited the gradient microstructure of B2 (Figure 5). However, it can be found that the size distribution of B2 particles has changed significantly after annealing as compared to the as-cast microstructure. The cold rolling can effectively lead to the formation of deformation shear bands within austenite grains. During the annealing process, new B2 particles can precipitate at deformation shear bands within unrecrystallized austenite grains [7]. Additionally, the total amount of reduction during cold rolling is large enough to provide nucleation sites for recrystallization during subsequent annealing at 900 °C. This will result in the formation of new B2 at the grain boundaries of recrystallized austenite as well [7]. Thus, the appearance frequency of B2 with a smaller size in an annealed sample can significantly increase no matter which layer is used as compared to an as-cast sample. Moreover, no B2 bands can be found in the present annealed steel, which is quite different from conventionally processed Ni-bearing Fe-Mn-Al-C lightweight steels [7,8]. The reason for this result is that subrapid solidification can effectively inhibit the formation of δ-ferrite [46,47], thus avoiding the formation of B2 through ordering.

Additionally, note that the austenite shows a gradient size in the as-annealed sample (Figure 8), which is quite different from conventionally processed Ni-bearing Fe-Mn-Al-C lightweight steels [7,8]. This is due to the fact that, compared with the quarter layer and center layer, austenite at the surface layer undergoes the largest deformation and has the highest deformation storage energy during cold rolling, and its large size indicates easier deformation [48], resulting in the higher-density shear bands inside austenite. Therefore, in the subsequent annealing process, the recrystallization nucleation rate of austenite at the surface layer is higher and the growth rate is faster. As a result, the recrystallized austenite grains meet with each other quickly, cannot continue to grow and eventually form smaller austenite grains at the surface layer. In contrast, the reduction in grain size and cold rolling deformation result in the formation of moderate austenite at the quarter layer and the largest austenite at the center layer.

### 4.2. Strain Hardening Behavior and its Influencing Factors

**Figure 10:**
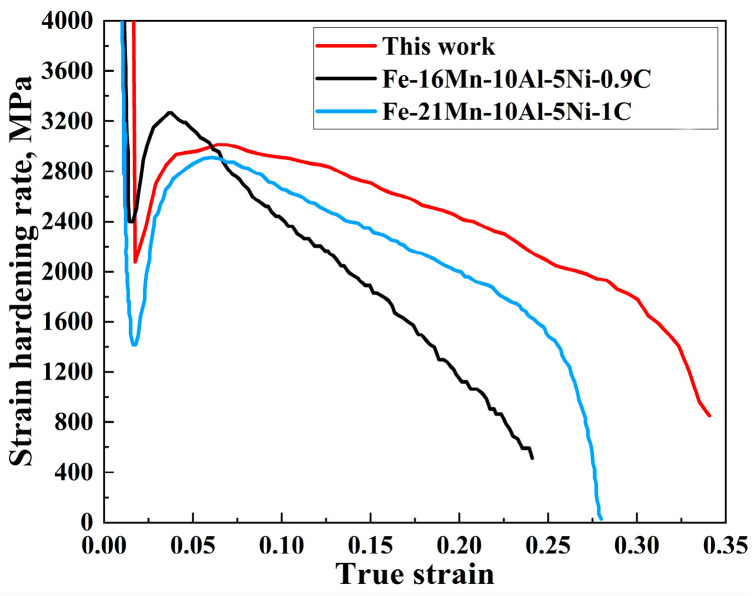
Strain hardening rate (SHR) curves of present steel and conventionally processed steels [13,35].

The strain hardening rate (SHR) of the present curves for annealed steel and conventionally processed steel is summarized in Figure 10 for comparison. These curves show three stages with varying trends in SHR. The rapid descent stage corresponds to the elastic deformation. The ascending stage corresponds to the yield plateau. Additionally, the subsequent slow descent stage corresponds to the uniform deformation. Note that the SHR of the present steel shows a much slower decline stage during uniform deformation than that of other conventionally processed Ni-containing Fe-Mn-Al-C lightweight steels, indicating a stronger strain hardening, which has been proven to be mainly responsible for the improvement of ductility [13,35].

**Figure 11:**
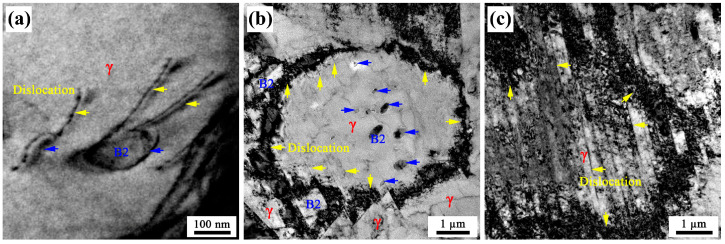
TEM micrographs showing deformation microstructures: (**a**) dislocations are pinned at B2-austenite interfaces (2%); (**b**) dislocations inside austenite grains and high-density tangled dislocations at grain boundaries (10%); (**c**) planar slip in austenite (20%).

In order to analyze the strain hardening behavior and clarify the reasons for the strong strain hardening effect of the present steel, the deformation microstructures after different strains were characterized by TEM (Figure 11). Figure 11a shows the dislocations are pinned and bowed out at B2-austenite interfaces after 2% straining, which is consistent with that reported by Kim et al. [7], indicating that the B2 particles within austenite are not sheared by gliding dislocations. The nonshearable nature of B2 particles is beneficial for the high strain hardening rate even at ultrahigh yield strength levels, resulting in ductile high-strength steels [7,49].

After 10% straining, the dislocations inside grains and high-density tangled dislocations at the grain boundary can be observed in coarse austenite (Figure 11b). This indicates that a long-range back stress can be produced in present steel during tensile deformation, which has also been observed in conventionally processed Fe-16Mn-10Al-0.86C-5Ni steel [50]. It has been reported that back stress is an important factor contributing to the strain hardening of duplex steels [8,50]. Due to the remarkable difference in yield strength between B2 and austenite, soft austenite will begin the plastic deformation first, while the strong B2 phase still remains elastic. This results in the mechanical incompatibility between already deformed austenite and elastic B2, so as to produce the strain gradient across austenite-B2 interfaces. Further, the geometrically necessary dislocations (GNDs) will be piled up and blocked by austenite-B2 phase boundaries [50,51] to induce back stress. Note that, unlike B2-austenite duplex steels, austenite as the matrix also shows the characteristics of gradient distribution (Figure 8), which will be conducive to achieving strong strain hardening. Due to the grain-size gradient and the dependence of yield strength on grain size, the plastic deformation in gradient metals gradually propagates from a soft coarse-grained layer to a hard fine-grained layer [52,53], leading to a deformation gradient. Moreover, it has been proven that the storage of GNDs helps coordinate deformation between adjacent small and large grains [54]. Therefore, it can be reasonably inferred that during the deformation process of present steel, due to the difference in grain size, the deformation sequence of austenite is coarse austenite at the center layer, medium austenite at the quarter layer and fine austenite at the surface layer. The multistage discontinuous austenite deformation will lead to continuous dislocation slip and accumulation to produce the long-range back stress, which can effectively delay the decrease in SHR [55]. Thus, the present steel shows a much stronger strain hardening effect than that in conventionally processed Ni-bearing Fe-Mn-Al-C lightweight steels with uniform microstructure. It has been demonstrated that strain-hardening can make a considerable contribution to the ductility of alloys and steels [8,50,51,56].

Furthermore, planar slip occurs in austenite grains after 20% straining (Figure 11c). The formation and intersection of the planar slip can result in high total elongation of Fe-Mn-Al-C steel due to a strong strain hardening effect [57,58,59]. The dislocations usually slip through the whole austenite grain, indicating their length scale is the same as the grain size [60]. Compared with fine-grained steel, the strain hardening by dislocation slip is saturated late in coarse-grained steel [61], implying a stronger strain hardening. Thus, the larger grain size (average: 4.52 µm) of austenite in the present steel results in better ductility than that of conventionally processed Ni-bearing Fe-Mn-Al-C lightweight steels. In addition, the high-density substructures can be regarded as impenetrable obstacles to the movement and further slip of dislocations, leading to high-strain hardening [62]. Additionally, the fine substructures can refine austenite grains and reduce the effective mean-free path of dislocations, resulting in strong strain hardening [63].

Consequently, the synergistic effects of B2 pinning dislocation, plane slip and much stronger back stress resulting from gradient duplex microstructure are responsible for the strong strain hardening of present steel. The most important microstructural characteristics of the present steel, which are different from the conventionally processed Ni-bearing Fe-Mn-Al-C lightweight steels, are the formation of a gradient duplex microstructure originating from the twin-roll strip casting technology. Thus, it can be inferred that the extremely heterogeneous gradient duplex microstructure is the most important reason for the much stronger strain-hardening effect of the present steel, thereby achieving superior strength–ductility balance.

### 4.3. Comparison of This Work with Other Conventionally Processed Steels

Compared with other conventionally processed Ni-containing Fe-Mn-Al-C lightweight steels, although the experimental steel has a slightly lower yield strength (760 MPa) and ultimate tensile strength (1147 MPa), its total elongation can reach as high as 43.2% (Figure 9a) [7,8,9,13]. The reasonable reasons for these results are as follows: On the one hand, austenite in the experimental steel shows a very large grain size (average: 4.52 µm), which inevitably leads to a decrease in yield strength. However, as mentioned above, the strong strain hardening effect resulting from the formation and intersection of planar slip within large austenite grains is beneficial for the high ductility. On the other hand, according to Kim [7] and Park [9], the intragranular B2 particles pinning dislocations can effectively improve the yield strength of Ni-containing Fe-Mn-Al-C lightweight steels. In this work, the cold rolling reduction (from 2.02 to 1 mm, 50.5%) is smaller than that in the references (>66.6%) [7,8,9]. The small cold rolling reduction cannot produce enough deformation shear bands within austenite grains to be the nucleation sites of B2. Thus, a lower fraction of intragranular B2 particles can precipitate in present steel than that in other conventionally processed steels, which cannot provide enough contribution to the yield strength.

## 5. Conclusions

In this work, the microstructure and tensile properties of Fe-16Mn-9Al-0.8C-3Ni steel manufactured by the twin-roll strip casting technology were investigated. Significantly different from conventionally processed Ni-bearing Fe-Mn-Al-C lightweight steels, a special gradient B2-austenite microstructure without B2 bands can be obtained due to the temperature gradient along the thickness direction. The present steel shows much stronger strain hardening, mainly due to the synergistic effect of B2 pinning dislocation, plane slip and strong back stress resulting from the gradient duplex microstructure. The multistage discontinuous austenite deformation and the mechanical incompatibility between austenite and B2 are believed to make the most important contributions to the strong strain hardening effect. Moreover, the coarse austenite grains (~4.52 µm) can also favor the improvement of ductility. As a result, the present steel achieves an ultra-high total elongation (43.2%) while ensuring an ultimate tensile strength higher than 1100 MPa. Such few processing steps and excellent mechanical properties make twin-roll strip casting technology an ideal alternative for the production of Ni-bearing Fe-Mn-Al-C lightweight steels.

## Data Availability

No new data were created or analyzed in this study. Data sharing is not applicable to this article.
